# Neural map formation in the mouse olfactory system

**DOI:** 10.1007/s00018-014-1597-0

**Published:** 2014-03-18

**Authors:** Haruki Takeuchi, Hitoshi Sakano

**Affiliations:** 1 Department of Brain Function, School of Medicine, University of Fukui, 23-3 Shimo-aizuki, Matsuoka, Fukui, 910-1193 Japan; 2 JST, PRESTO, 4-1-8 Honcho, Kawaguchi, Saitama, 332-0012 Japan; 3 Department of Biophysics and Biochemistry, Graduate School of Science, University of Tokyo, 2-11-16 Yayoi, Bunkyo-ku, Tokyo, 113-0032 Japan

**Keywords:** Olfactory map, Olfactory sensory neurons, Axonal projection, Odorant receptors, G-protein-coupled receptors, Agonist-independent activity, cAMP

## Abstract

In the mouse olfactory system, odorants are detected by ~1,000 different odorant receptors (ORs) produced by olfactory sensory neurons (OSNs). Each OSN expresses only one functional OR species, which is referred to as the “one neuron–one receptor” rule. Furthermore, OSN axons bearing the same OR converge to a specific projection site in the olfactory bulb (OB) forming a glomerular structure, i.e., the “one glomerulus–one receptor” rule. Based on these basic rules, binding signals of odorants detected by OSNs are converted to topographic information of activated glomeruli in the OB. During development, the glomerular map is formed by the combination of two genetically programmed processes: one is OR-independent projection along the dorsal–ventral axis, and the other is OR-dependent projection along the anterior-posterior axis. The map is further refined in an activity-dependent manner during the neonatal period. Here, we summarize recent progress of neural map formation in the mouse olfactory system.

## Introduction

**Fig. 1:**
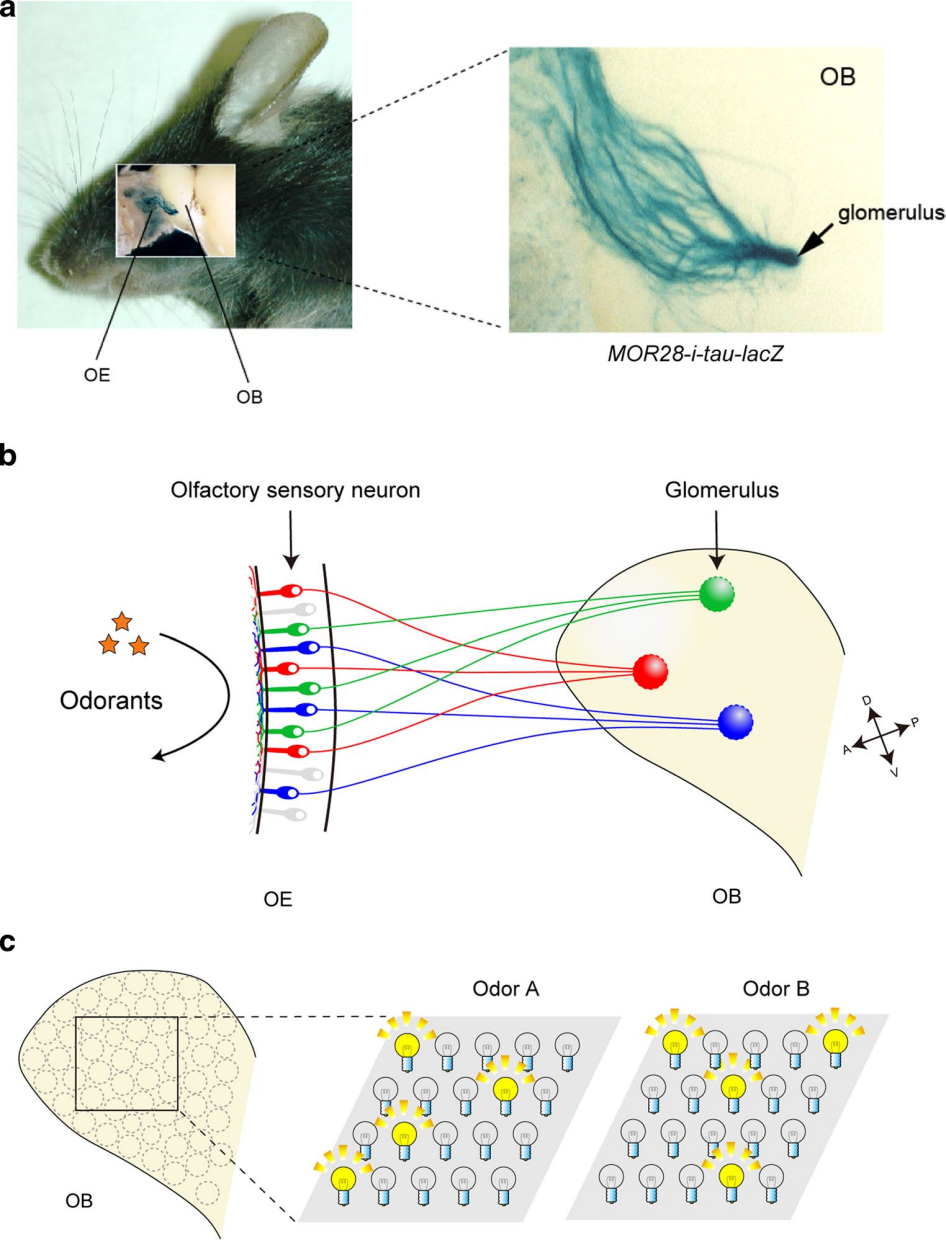
The mouse olfactory system. **a** MOR28-expressing OSN axons are shown in* blue* stained with X-gal in the transgenic mouse containing *MOR28*-*ires*-*tau*-*lacZ*. OSNs expressing the *MOR28* transgene converge their axons to a specific glomerulus in the OB (indicated by an *arrow*). **b** In the OE, each OSN expresses only one functional OR gene in a monoallelic manner. Furthermore, OSN axons expressing the same OR species target to a specific site in the OB, forming a glomerular structure. **c** Odor signals received in the OE are converted to a topographic map of activated glomeruli in the OB. *OE* olfactory epithelium, *OB* olfactory bulb, *D* dorsal, *V* ventral, *A* anterior, *P* posterior

**Fig. 2:**
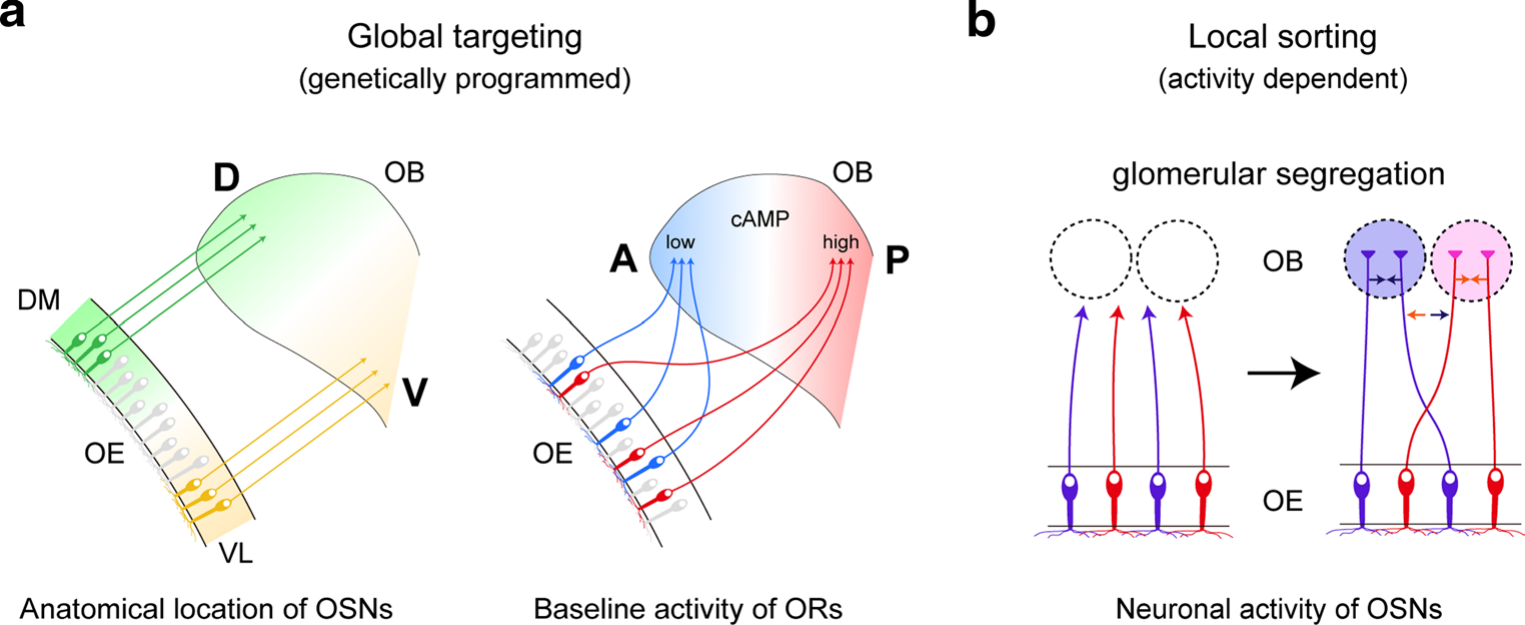
Stepwise regulation of olfactory map formation. **a** OSN axons are guided to approximate destinations in the OB by a combination of D–V patterning and A–P patterning. D–V projection is regulated by the anatomical locations of OSNs within the OE. A–P projection is achieved through cAMP signals induced by agonist-independent OR activities. These processes, forming a coarse map topography, are genetically programmed and independent of the neuronal-activity of OSNs. **b** During the neonatal period, the map is further refined in an activity-dependent manner. Glomerular segregation occurs via adhesive and repulsive interactions of neighboring axons. *DM* dorsomedial, *VL* ventrolateral, *D* dorsal, *V* ventral, *A* anterior, *P* posterior, *OE* olfactory epithelium, *OB* olfactory bulb

In the mouse, various odorants are detected with approximately 1,000 different odorant receptors (ORs) expressed in the olfactory sensory neurons (OSNs) [1]. Each OSN in the olfactory epithelium (OE) expresses only one functional OR gene in a mono-allelic manner [2]. Furthermore, OSNs expressing the same OR converge their axons to a specific pair of glomeruli at stereotyped locations in the olfactory bulb (OB) (Fig. 1a, b) [3]. Thus, the odor information detected in the OE is topographically represented as the pattern of activated glomeruli in the OB (Fig. 1c) [4]. A remarkable feature of OSN projection is that ORs play instructive roles in projecting OSN axons to the OB. For dorsal–ventral (D–V) projection, positional information of OSN cells within the OE regulates both OR gene choice and expression levels of axon guidance molecules, e.g., Neuropilin-2 (Nrp2) and Semaphorin-3F (Sema3F), thus correlating the OR identity to the glomerular location along the D–V axis (Fig. 2a, left) [5, 6]. Unlike D–V projection, anterior–posterior (A–P) projection is independent of the positional information of OSN cells, but instead dependent on the expressed OR species (Fig. 2a, right). We have previously found that both global targeting along the A–P axis and local sorting of OSN axons for glomerular segregation are regulated by OR-derived cAMP as a second messenger [7]. In the OB, A–P projection molecules such as Neuropilin-1 (Nrp1) and Plexin-A1 (PlxnA1) are detected on axon termini of OSNs, forming a complementary gradient in a glomerular map [8]. OR-derived cAMP signals also regulate the expression of glomerular segregation molecules, e.g., Kirrel2 and Kirrel3, for olfactory map refinement in a neuronal activity-dependent manner (Fig. 2b) [9]. Unlike A–P projection molecules, glomerular segregation molecules show mosaic distribution in the glomerular map. Naris occlusion experiment indicated that stimulus-driven neuronal activity contributes to the local sorting of OSN axons, but not to global targeting along the A–P axis, which is not affected by odor ligands [10]. How do the expressed OR molecules regulate A–P targeting of OSN axons and glomerular segregation? What are the sources of the cAMP signals, and how are the signals generated? Here, we overview the recent progress in the neural map formation in the mouse olfactory system.

### OSN projection along the D–V axis

**Fig. 3:**
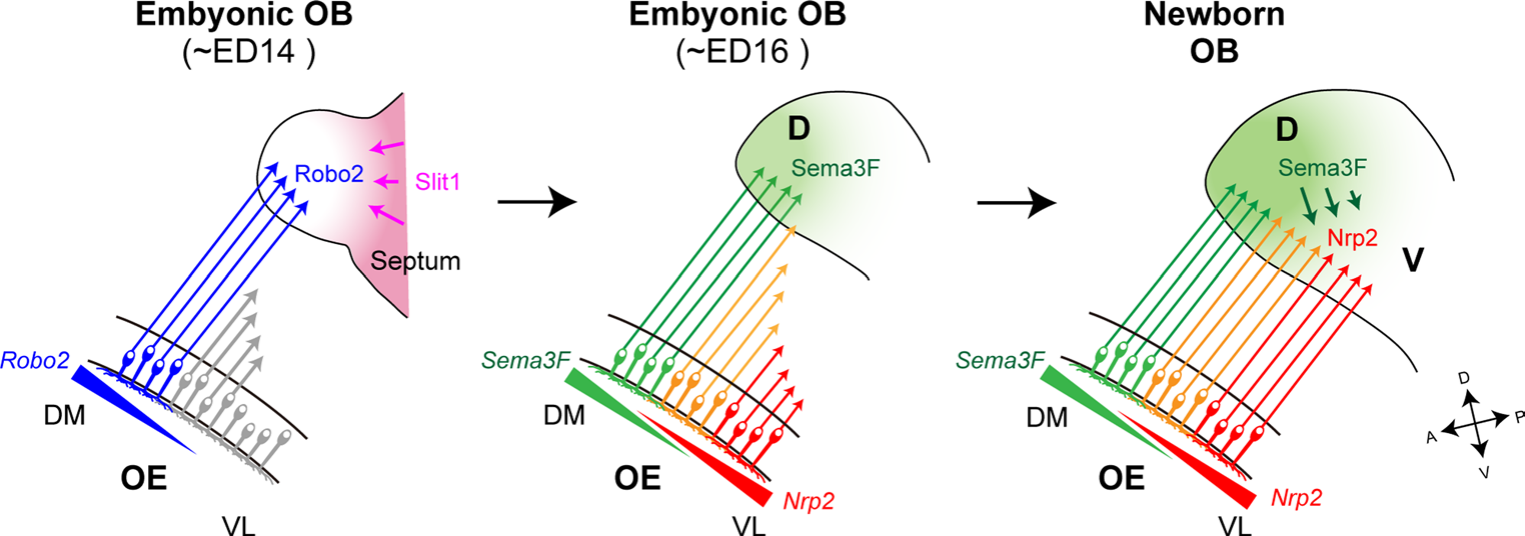
A model for axonal projection of OSNs along the D–V axis. In the OE, D-zone OSNs mature first and extend their axons to the OB earlier than V-zone OSNs. Early arriving D-zone axons express Robo2 and project to the prospective anterodorsal domain of the OB with the aid of repulsive interactions with Slit1 expressed in the septum and ventral OB at early developmental stages (*left*). In OSNs, an axon guidance receptor, Nrp2, and its repulsive ligand, Sema3F, are expressed in a complementary and graded manner. Sema3F is secreted in the anterodorsal region of the OB by early arriving D-zone axons (*middle*). Axonal extension of OSNs occurs sequentially along the DM–VL axis of the OE as the OB grows ventrally during embryonic development. This sequential projection helps to maintain topographic order during the process of axonal projection. Sema3F secreted by the D-zone axons in the OB prevents the late-arriving Nrp2^+^ axons from invading the dorsal region of the OB (*right*). *DM* dorsomedial, *VL* ventrolateral, *D* dorsal, *V* ventral, *A* anterior, *P* posterior, *ED* embryonic day

For OSN projection along the D–V axis, there is a close correlation between the anatomical locations of OSNs in the OE and their axonal projection sites in the OB (Fig. 2a, left) [11]. The preservation of the spatial relationship of cell bodies and their axonal target sites is widely seen in other brain systems including the visual system [12–14]. In the mouse olfactory system, two sets of repulsive signaling systems, Nrp2/Sema3F and Robo2/Slit1, have been reported to participate in the D–V projection of OSNs [15–17]. D-zone axons expressing a guidance receptor, Robo2, navigate to the D domain of the OB through the repulsive effects of the Slit ligands expressed in the V domain of the OB (Fig. 3, left) [16]. These molecules are assumed to contribute to the separation of D and V domains [6, 16]. In the OE, OR genes expressed by OSNs that project to the D domain of the OB are distributed throughout the D zone [18]. However, V-zone-specific OR genes exhibit spatially limited expression. Each OR gene possesses its unique expression area, which is distributed in an overlapping and continuous manner along the dorsomedial–ventrolateral (DM–VL) axis of the OE [5]. The reliability of the relationship between D–V positioning of glomeruli and DM–VL locations of OSNs has been demonstrated by DiI staining and in situ hybridization. How is this positional information of neurons in the OE translated to their target sites in the OB during olfactory map formation? Nrp2 is expressed on OSN axons in such a way to form a gradient in the OB along the D–V axis. Loss-of-function and gain-of-function experiments demonstrated that Nrp2 indeed regulates the axonal projection of OSNs along the D–V axis [6]. Based on the visual system, the repulsive ligand, Sema3F, was expected to be produced by the cells in the target OB. Curiously, however, Sema3F transcripts were detected in the OE but not in the OB. Animals in which Sema3F expression was specifically blocked in OSNs showed mistargeting of Nrp2^+^ axons along the D–V axis [6]. These findings indicate that, in the olfactory system, an axon guidance receptor, Nrp2, and its repulsive ligand, Sema3F, are both expressed by OSN axons to regulate D–V projection (Fig. 3, right).

Expression levels of D–V targeting molecules, such as Nrp2 and Sema3F, are closely correlated with the expressed OR species. However, the transcription of Nrp2 and Sema3F is not downstream of OR signaling [10]. It has been reported that OR gene choice is not purely stochastic for D–V targeting and is dependent upon anatomical location in the OE [5]. This idea was demonstrated using transgenic mice in which the coding sequence of the transgenic OR gene is deleted and replaced by green fluorescent protein, GFP [19]. In these mice, the choice of the secondary OR gene in GFP-positive OSNs was not random, and primarily limited to a group of OR genes whose expression areas and transcription levels of the Nrp2 are comparable to those of the coding-deleted OR gene. If D–V guidance molecules are not regulated by OR-derived signals, how are their expression levels determined and correlated with the expressed OR species? We assume that both OR gene choice and Nrp2 expression levels are commonly regulated by positional information within the OE. This regulation is likely determined by cell lineage, resulting in the use of specific sets of transcription factors, which can explain the anatomical correlation along the DM–VL axis of the OE.

### Sequential projection of OSN axons

Using molecular markers for mature and immature OSNs, it has been shown that OSNs in D zone mature earlier than those in V zone during development [20]. Glomerular structures first emerge in the anterodorsal domain of the OB. These observations point toward an intriguing possibility that a repulsive ligand, Sema3F, is secreted by early arriving D-zone axons and is deposited in the anterodorsal OB to serve as a guidance cue to repel late-arriving V-zone axons that express Nrp2 receptor [6]. Then, what guides pioneer OSN axons to the anterodorsal area acting as a land mark? Robo2^+^ D-zone axons project to the dorsal region of the OB by repulsive interactions with secreted ligands [16, 21]. One of the Robo2 ligands, Slit1, is detected in the septum and ventral OB during early developmental stages. In the KO for the Robo/Slit system, OSN axons mistarget to surrounding non-OB tissues [21]. These observations suggest that repulsive interactions between Robo2 and Slit1 are needed to restrict the first wave of OSN projection to the anterodorsal OB (Fig. 3, left). During development, the glomerular map expands ventrally and the embryonic OB represents the prospective dorsal OB. Axonal projection of OSNs occurs sequentially from the DM to the VL area in the OE, which helps to establish the map order in the OB along the D–V axis (Fig. 3, middle). Spatiotemporal regulation of axonal projection of OSNs aided by Robo2 and Slit1, and the graded expression of Nrp2 and Sema3F helps in establishing the topographic order of the olfactory map along the D–V axis [6].

### OR-instructed OSN projection along the A–P axis

It is well established that each OSN expresses only one functional OR gene in a mono-allelic manner [2]. Furthermore, the olfactory map is comprised of discrete glomeruli, each representing a single OR species [3, 22, 23]. The instructive role of the OR protein in OSN projection was demonstrated by the coding-swap experiments of OR genes [24, 25]. Since OSNs expressing the same OR are scattered in the OE for A–P targeting, topographic organization must occur during the process of axonal projection to the OB (Fig. 2a, right). Unlike neural map formation in other sensory systems where relative positional information is preserved between the periphery and the brain, there is no such correlation for the projection along the A–P axis in the mouse olfactory system. Based on the observation that OR molecules are detected not only on cilia but also in axon termini of OSNs [25–27], it was once thought that the OR protein itself may act as axon guidance receptors detecting the target cues in the OB and also mediate homophilic interactions among “like” axons [28]. Although these models were attractive, recent studies argue against them. Instead of directly acting as guidance receptors or axon sorting molecules, ORs appear to regulate transcription levels of A–P targeting and glomerular segregation molecules by OR-derived cAMP signals whose levels are uniquely determined by the OR species [8, 29].

We hypothesized that each OR species generates a unique level of cAMP that regulates expression of axon-guidance molecules, e.g., Nrp1 and its repulsive ligand Sema3A. It was found that OSNs producing high levels of cAMP project their axons to the posterior OB, while those producing low levels target the anterior OB (Fig. 2a, right) [8]. When protein levels of Nrp1 were measured in axon termini of OSNs, Nrp1 was found in an anterior-low/posterior-high gradient in the OB. Increases or decreases of Nrp1 expression in OSNs caused posterior or anterior glomerular shifts, respectively [30]. Furthermore, the A–P topography of the glomerular map was perturbed in mice deficient for Nrp1 or Sema3A. How do the axon guidance molecules regulate the topographic order of the olfactory map along A–P axis? Surprisingly, we found that map order emerges in axon bundles, well before they reach the target [30]. It appears that pretarget axon sorting plays an important role in the organization of the olfactory map. Neuropilin-1 and Sema3A are both expressed in OSNs, but in a complementary manner. Within the axon bundles, Nrp1-low/Sema3A-high axons are sorted to the central compartment of the bundle, whereas Nrp1-high/Sema3A-low axons are confined to the outer-lateral compartment. OSN-specific KO of Nrp1 or Sema3A not only perturbed axon sorting within the bundle, but also caused an anterior shift of glomeruli in the OB [30]. These results indicated that pretarget axon sorting within the bundle contributes to the olfactory map formation along the A–P axis.

### Activity-dependent glomerular segregation

During embryonic development, a coarse map topography is established by a combination of D–V patterning, based on anatomical locations of OSNs, and A–P patterning, based on OR-derived cAMP signals (Fig. 2a). However, in the newborn animal, neighboring glomerular structures are intermingled before birth, and discrete glomeruli emerge during the neonatal period. After OSN axons reach their approximate destinations in the OB, further refinement of the glomerular map needs to occur through fasciculation and segregation of axon termini in an activity-dependent manner (Fig. 2b). To study how OR-specific axon sorting is controlled, we searched for a group of genes whose expression profiles correlate with the expressed OR species. Using the transgenic mouse in which the majority of OSNs express a particular OR, such genes were indeed identified: they include those that code for homophilic adhesive molecules, e.g., Kirrel2 and Kirrel3 [9]. Mosaic gain/loss of function of these genes generated duplicated glomeruli even though the expressed OR species were the same, suggesting that Kirrel2 and Kirrel3 play a role in the attraction of “like” OSN axons. Repulsive molecules, such as ephrinAs and EphAs, are also expressed in a complementary and OR-specific manner in each subset of OSNs [9]. Therefore, interactions between two subsets of axons, one that is ephrinA-high/EphA-low and the other that is ephrinA-low/EphA-high, may be important for the segregation of “non-like” OSN axons. We assume that a specific set of adhesive and repulsive molecules, whose expression levels are determined by OR molecules, regulate the axonal fasciculation of OSNs. It is not clear at this point how many sets of sorting molecules are involved in glomerular segregation. However, several sets of adhesion/repulsion molecules should be enough to segregate neighboring glomerular structures.

Unlike the global targeting of OSN axons in embryos, local sorting appears to occur in an activity-dependent manner in the neonatal animals. Blocking neuronal activity by the overexpression of an inward rectifying potassium channel, Kir2.1, severely affects glomerular segregation [10, 31]. Mice that are mosaic KO for CNGA2, a component of CNG channels, reveal segregation of CNGA2-positive and -negative glomeruli for the same OR [9, 32]. The expression levels of glomerular segregation molecules are affected by the CNGA2 mutation in OSNs. In the CNGA2 KO, Kirrel2 was downregulated while Kirrel3 was upregulated, indicating that these genes are transcribed in an activity-dependent manner [9]. Interestingly, expression levels of glomerular segregation molecules (e.g., Kirrel2 and Kirrel3) were affected by unilateral naris occlusion. In the occluded naris, Kirrel2 expression was downregulated and Kirrel3 expression was upregulated, whereas expression of A–P targeting molecules, Nrp1 and PlxnA1, was not affected [10]. These results indicate that stimulus-driven OR activity contributes to the local sorting of OSN axons but does not affect global targeting along the A–P axis.

We examined whether odorous stimuli can change the expression profile of A–P targeting and glomerular segregation molecules [10]. In transgenic mouse, in which the *MOR29B* gene is tagged with *IRES*-*gap*-*EYFP*, were housed in the presence of vanillin, a ligand for MOR29B. In control mice not exposed to vanillin, MOR29B glomeruli ranked in the 30th percentile from the lowest Kirrel2-staining intensity among ~300 glomeruli analyzed. In contrast, when the mice were exposed to vanillin, the ranking went up to 60th percentile from the lowest. Interestingly, Nrp1 expression was not affected by vanillin exposure in the MOR29B-positive OSNs. These results indicate that the expression of glomerular segregation molecules is regulated by ligand-induced OR signals, whereas the expression of A–P-targeting molecules is likely to be driven by ligand-independent OR activity [10].

### Ligand-independent OR activity regulates A–P targeting

**Fig. 4:**
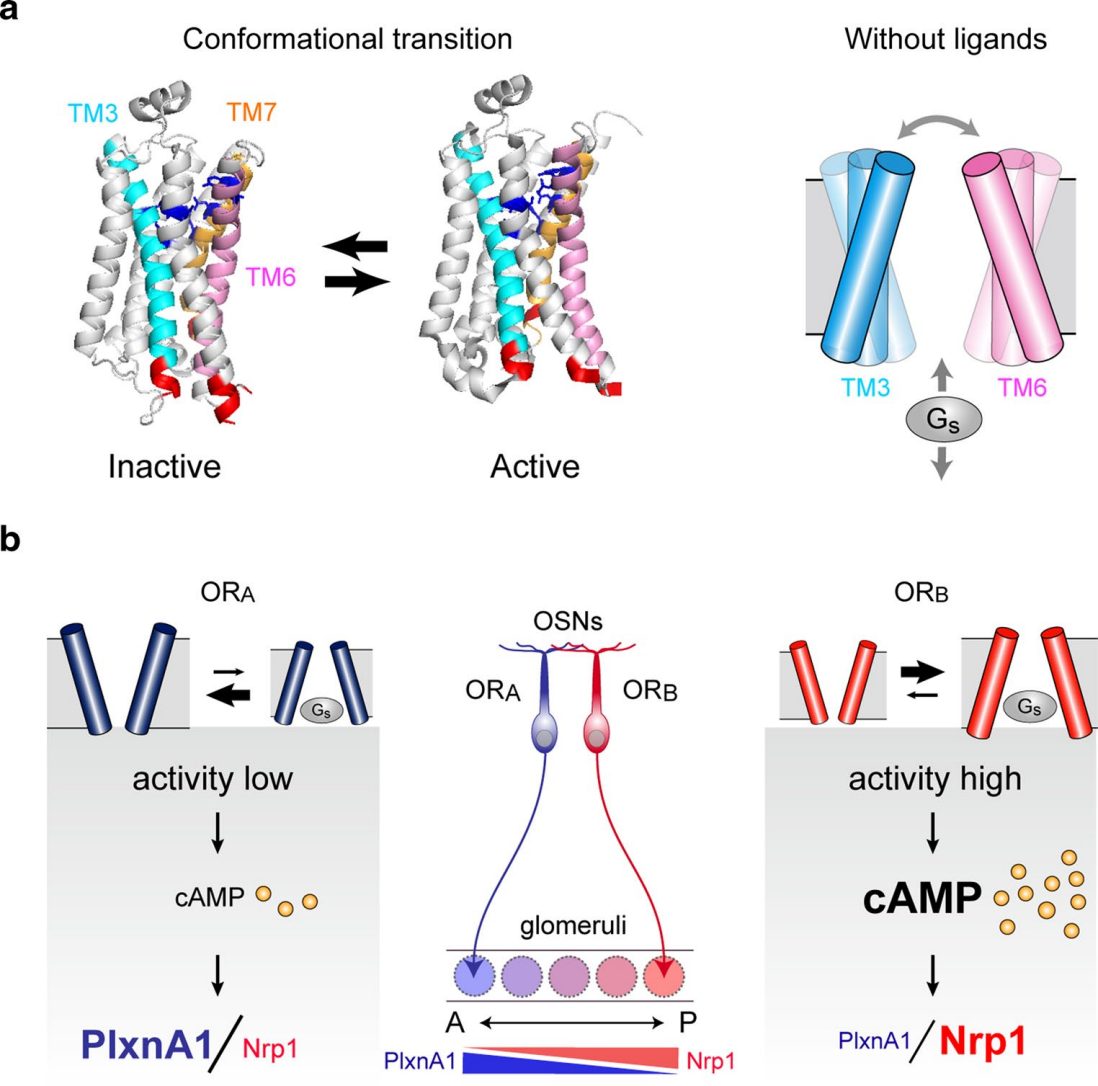
**a** Conformational changes of GPCRs (modified from Ref. [39] by Rasmussen et al.). G-protein-coupled receptors (GPCRs) are known to possess two different conformations, active and inactive. In the absence of ligands, GPCRs spontaneously interchange between these conformations, generating agonist-independent baseline activity (*right*). **b** Each OR possesses a unique level of baseline activity and generates a specific amount of cAMP using G_s_, but not G_olf_. The levels of cAMP signals are converted to transcription levels of A–P targeting molecules, e.g., Nrp1 and PlxnA1. Activity-high axons project to the posterior region of the OB, whereas activity-low axons project to the anterior OB. *TM* transmembrane domain, *OR* odorant receptor

As described above, OR-derived signals that regulate A–P targeting molecules are not affected by extrinsic stimuli, including odor ligands. Then, what kind of OR activity could be responsible for the regulation of A–P targeting, and how is it generated? G-protein-coupled receptors (GPCRs), including ORs, are known to possess two different conformation states, active and inactive (Fig. 4a, left) [33]. After birth, in the presence of ligands, agonists stabilize the receptor in an active form, whereas inverse agonists lock it in an inactive form. During embryonic development, in the absence of ligands, GPCRs spontaneously flip between active and inactive conformations producing a baseline level of cAMP (Fig. 4a, right). For different OR species, variable but specific levels of baseline activities have been reported [34]. However, the agonist-independent activity had long been considered to be noise created by GPCRs, and its functional role was not fully appreciated. Since naris occlusion did not affect the expression of A–P targeting molecules, we hypothesized that the ligand-independent OR activity may participate in the regulation of A–P targeting of OSN axons. In order to examine this possibility, we attempted to generate the activity mutants of ORs. The initial experiment with ORs was not successful due to the challenges of achieving adequate membrane expression in the heterologous system. In addition, due to the vast diversity of OR family proteins and the lack of three-dimensional (3D) structural information, prediction and screening of activity mutants were difficult for OR molecules.

In contrast, β2-adrenergic receptor (β2-AR), a GPCR with the highest sequence homology to ORs, is easier to express in transfected cells and shares many functional similarities with ORs. When expressed in OSNs with the OR gene promoter, β2-AR maintains the one neuron-one receptor rule, couples with the α subunit of G_s_ or G_olf_, and substitutes ORs for receptor-instructed axonal projection [35]. Furthermore, β2-AR has advantages of being well characterized for distinct receptor functions. Based on mutational studies, the key amino acid residues in the β2-AR that are required for G protein coupling, ligand binding, and the generation of agonist-independent activity are well characterized [36–38]. Recently, the 3D structures of β2-AR in its active state and in a complex with a stimulatory G protein, G_s_, have been determined [39, 40]. As a result of these favorable features, we selected β2-AR for the transgenic analysis of the agonist-independent GPCR activity in axonal projection of OSNs [10]. Among the β2-AR mutations analyzed, some affected both agonist-independent and dependent activities, whereas others affected only one of the two activities. We predict that mutations affecting G-protein activation would alter both agonist-dependent and -independent activities, whereas those altering ligand interactions would change agonist-dependent activity. Mutations that affect conformational transitions should alter the agonist-independent receptor activity. For further transgenic studies, we selected three β2-AR mutants from the collection, which significantly altered agonist-independent receptor activity, but not the agonist-dependent activity.

We generated transgenic mice expressing the mutant or wild-type (WT) β2-AR using an OR gene promoter [10]. This was performed by replacing the *MOR23* coding sequence with that of β2-AR in the *MOR23* transgenic minigene cassette. The glomerular locations were studied for the WT and mutant β2-ARs tagged with different fluorescent markers. The activity-low β2-AR mutants generated glomeruli anterior to that of the WT. In contrast, the activity-high mutation, caused a posterior shift of glomeruli. This finding showed a good correlation between the agonist-independent activities of β2-ARs and their corresponding glomerular locations in the OB along the A–P axis. We also examined the expression levels of A–P-targeting molecules in the β2-AR glomeruli [10]. Neuropilin-1 expression levels in β2-AR-expressing OSNs were increased by the activity-high mutation, but lowered by the activity-low mutations. Expression levels of PlxnA1 were also affected by the activity mutations, however, the results were inverse compared with those of Nrp1, because PlxnA1 expression is inversely regulated by cAMP signals. It is notable that expression levels of glomerular segregation molecules, e.g., Kirrel2 and Kirrel3, were not affected at all by the activity mutations indicating that glomerular segregation molecules are regulated by distinct OR signals.

To examine whether the correlation between the agonist-independent activities and glomerular locations holds true for natural ORs, we performed the following experiments [10]. We dissected the mouse OB into three sections: anterior, middle, and posterior. Thirty OR genes were cloned, transfected into HEK293 cells, and analyzed for their activities without ligands by the luciferase reporter assay [41]. ORs cloned from the anterior OB produced relatively lower levels of agonist-independent activities, and those from the posterior OB generated higher levels. Thus, the agonist-independent OR activity appears to be the major determinant of expression levels of A–P targeting molecules (Fig. 4b). It should be noted that for natural ORs, the promoter activities, protein stabilities, and membrane transport could be potential factors affecting total cAMP signal levels. However, at least among the ORs analyzed in our study, differences in cell-surface expression levels were within 15 %.

### Differential usage of G_s_ and G_olf_ in OSNs

Our studies demonstrated that OR-instructed A–P targeting and glomerular segregation are differentially regulated by two distinct OR-derived cAMP signals. How are these two types of regulation separately controlled during development? To address this question, we analyzed the onset of expression for various genes involved in axon guidance and signal transduction in OSNs [10]. At embryonic day (E)13.5, hybridization signals were detected for A–P-targeting molecules (e.g., Nrp1), but not for glomerular segregation molecules (e.g., Kirrel2). Kirrel2 expression became prominent only at the late stage of embryonic development. We also analyzed the onset of G_s_, G_olf_, and other signal transduction molecules. Hybridization signals were detected for G_s_ at E13.5. In contrast, G_olf_ is expressed at E17.5, but not at E13.5, indicating that G_olf_ is not required for the expression of A–P-targeting molecules.

**Fig. 5:**
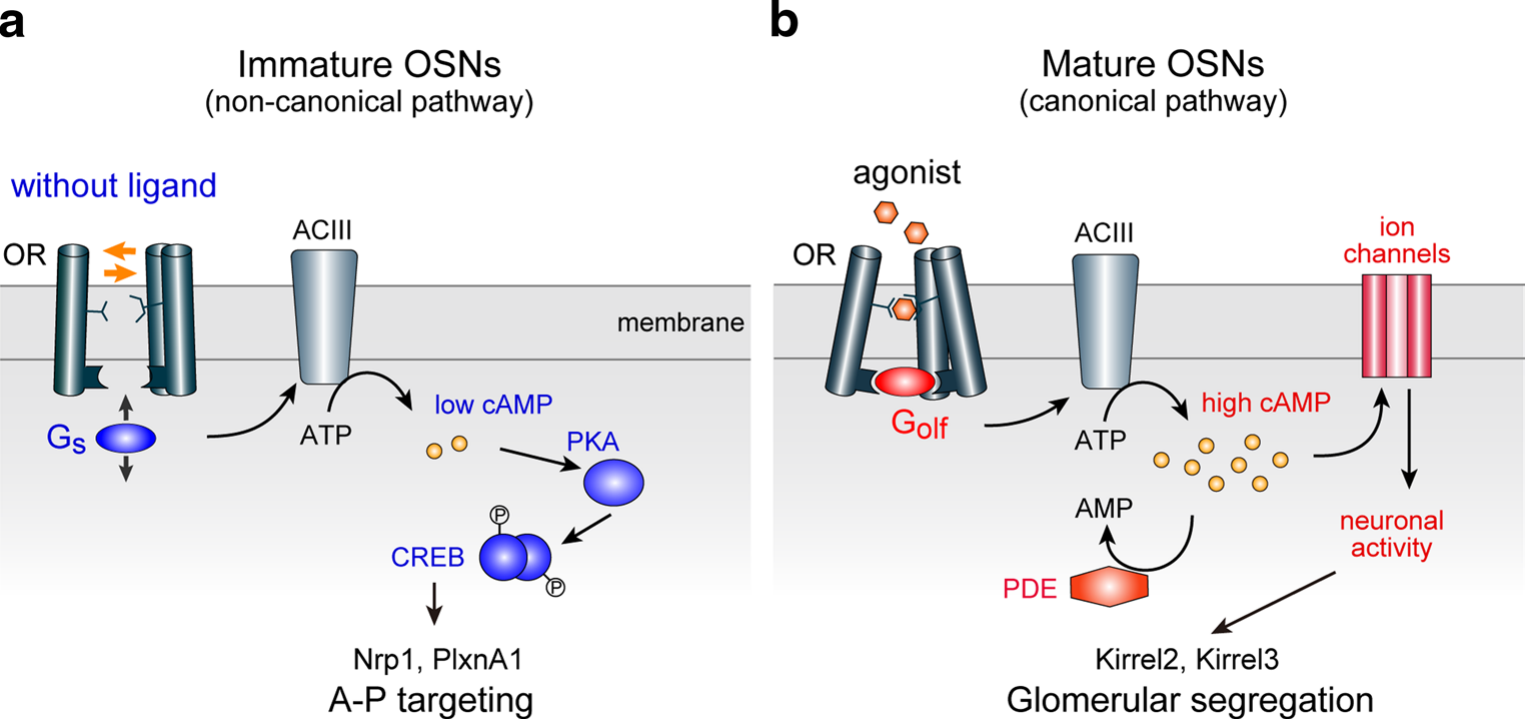
A two-step model for OR-instructed axonal projection of OSNs. OR-instructed A–P targeting and glomerular segregation are differentially regulated by distinct OR-derived cAMP signals. **a** A–P targeting is regulated by agonist-independent OR activity using a non-canonical signaling pathway. In immature OSNs, each OR generates a unique level of cAMP by agonist-independent baseline activity via G_s_ and ACIII. The level of cAMP signals is converted to transcription level of A–P targeting molecules, e.g., Nrp1 and PlxnA1, through the cAMP-activated PKA pathway, phosphorylating the transcription factor CREB. **b** Glomerular segregation is regulated by stimulus-driven neuronal activity using a canonical signal transduction pathway. In mature OSNs, different ORs generate different levels of neuronal activity using extrinsic stimuli, which ultimately determine the transcription levels of glomerular segregation molecules, e.g., Kirrel2 and Kirrel3. *OR* odorant receptor, *AC* adenylyl cyclase, *PKA* protein kinase A, *CREB* cAMP responsive element binding protein, *PDE* phosphodiesterase

G_s_ and G_olf_ are structurally similar, sharing 88 % amino acid identity, and both mediate OR signals, activating adenylyl cyclase type III (ACIII) in OSNs. However, their functional differences in the cellular context were not fully recognized. What could be the reason that G_s_ and G_olf_ are differentially expressed in OSNs during development? We examined the biochemical properties of G_s_ and G_olf_ in mediating OR signals [10]. We generated G_s_ or G_olf_ fusion proteins for different ORs, whose agonists have been established. Both agonist-independent and -dependent cAMP signals were measured in vitro by the dual-luciferase assay using β2-AR as a control. We detected much higher agonist-independent cAMP signals with G_s_ than with G_olf_, whereas ligand response properties were similar between G_s_ and G_olf_. We concluded that G_s_ mediates agonist-independent activity more efficiently than G_olf_. This notion was further confirmed by the loss-of-function experiments using knockout (KO) mice of G_s_ and G_olf_ [10]. It was found that expression of A–P targeting molecules was affected by the G_s_ conditional KO, whereas the glomerular segregation molecules were unaffected. In contrast, G_olf_ KO affected the glomerular segregation, but not A–P targeting. Taken together, our results demonstrated that G_s_ plays a major role in regulating A–P targeting in immature OSNs, followed by the role of G_olf_ for glomerular segregation in mature OSNs (Fig. 5).

## Conclusions

Our recent studies revealed that A–P targeting and glomerular segregation molecules are separately regulated by distinct signals of ORs, even though both are using OR-derived cAMP as a second messenger. Glomerular segregation is regulated by stimulus-driven neuronal activity, whereas A–P targeting is regulated by agonist-independent baseline activity of ORs [10]. How these two types of signals are separately transduced can be explained by the following. One mechanism would be temporal insulation: Different OR signals are processed for cAMP production at different stages of olfactory development. Our studies indicated that cAMP signals for A–P projection and glomerular segregation are separately processed with distinct signal transduction molecules at immature and mature stages, respectively (Fig. 5). Differences between the two types of regulation may also be due to the subcellular localization of ORs, namely, cilia in mature OSNs vs. axon termini in immature OSNs.

Although spatial and insulation of two distinct OR signals may be important for the differential regulation, the difference in the source of cAMP signals appears to be the major basis for the difference in the distribution of A–P targeting (graded) and glomerular segregation (mosaic) molecules in the glomerular map. Our study demonstrated that the equilibrium of conformational transition of GPCRs without ligands determines the steady-state levels of cAMP in immature OSNs, which ultimately determine the expression levels of A–P-targeting molecules (Figs. 4b, 5a). In contrast, expression of glomerular segregation molecules is regulated by the stimulus-driven neuronal activity in mature OSNs. Amounts of stimuli appear to be the major determinant of the expression levels of glomerular segregation molecules (Fig. 5b). Thus, OR-specific rate-limiting factors of cAMP production are different between the agonist-independent and -dependent processes.

Until recently, GPCR studies had been focused on their ligand-dependent functions. As the baseline activity had long been considered to be noise created by GPCRs, its biological role was not fully appreciated. However, recent studies of crystal structures of β2-AR [39, 40] have revealed the inner workings of various GPCRs: The extracellular cavity determines ligand specificity and firing rates, whereas the intracellular cavity determines the G protein selectivity and levels of baseline activities. The olfactory system makes use of extensive functionality of the largest family of GPCRs: G_s_ utilizes intracellular diversity of ORs for axonal wiring specificity during development, whereas G_olf_ utilizes extracellular diversity to detect various environmental stimuli after birth and also to regulate olfactory map refinement.

After 22 years since the discovery of the OR-gene by Buck and Axel [1], it is now clear what defines the identity of OSNs in OR-instructed axonal projection. Our studies have revealed that the equilibrium of conformational transitions set by each OR is what determines the transcription levels of A–P targeting molecules in OSNs (Fig. 4) [10].
